# Paederoside, an active metabolite of Paederia scandens, alleviates osteoporosis by modulating Wnt/β-catenin signaling

**DOI:** 10.3389/fphar.2025.1670279

**Published:** 2025-09-24

**Authors:** Zhize Liu, Weitao He, Jingwei Zhang, Bo Su, Jiang Mu, Xintao Wang

**Affiliations:** ^1^ Department of Orthopedic Surgery, The Second Affiliated Hospital of Harbin Medical University, Harbin, China; ^2^ Department of Orthopedic Surgery, Affiliated Central Hospital of Dalian University of Technology, Dalian, Liaoning, China; ^3^ Department of Hand and Foot Surgery, Shandong Provincial Hospital Affiliated to Shandong First Medical University, Jinan, China

**Keywords:** *P. scandens* (Lour.) Merr. (*P. scandens*), paederoside, osteoporosis, Wnt/β-catenin sig-naling pathway, network pharmacology

## Abstract

**Introduction:**

*Paederia scandens* (Lour.) Merr. (*P. scandens*), a widely consumed edible wild botanical drug in East Asia, has traditionally been used in folk medicine for its purported health-promoting effects, including the relief of fractures, rheumatoid arthritis, and pain. Despite its common use, the bioactive metabolites and underlying mechanisms of action remain insufficiently understood. This study investigates the pharmacological effects and molecular mechanisms of *P. scandens* in the context of osteoporosis.

**Methods:**

An ovariectomized (OVX) rat model of osteoporosis was used to evaluate the effects of *P. scandens* extract on bone health. Network pharmacology analysis identified candidate metabolites related to osteoblast differentiation. Paederoside, the primary plasma-absorbed metabolite, was validated in OVX rats via HPLC-Q-TOF-MS. *In vitro* experiments assessed osteogenesis and osteoclast activity using an osteoblast-osteoclast co-culture system, while *in vivo* studies evaluated serum calcium and phosphorus levels, bone morphology, and bone mass.

**Results:**

*P. scandens* extract significantly improved bone mass, bone mineral density (BMD), and the trabecular bone microstructure in the OVX rat model. Paederoside promoted osteogenesis and suppressed osteoclast activity *in vitro*. *In vivo*, paederoside demonstrated pronounced anti-osteoporotic effects, as evidenced by elevated serum calcium and phosphorus levels, improved bone morphology, and increased bone mass. Paederoside upregulated osteogenesis-related markers (BMP2, Smad1, Col1a1, Bglap, Osterix, and Runx2) and downregulated osteoclastogenesis-related markers (RANKL, NFATc1, Acp5, Ctsk, and Mmp9). Transcriptomic analysis revealed that paederoside modulated the Wnt/β-catenin signaling pathway, inducing Wnt3a expression, inhibiting GSK-3β and β-catenin phosphorylation, and upregulating downstream targets such as Cyclin D1 and c-Myc.

**Discussion:**

In conclusion, *P. scandens* exerts protective effects against osteoporosis, and paederoside, identified as a primary plasma-absorbed metabolite, enhances osteoblastogenesis and suppresses osteoclastogenesis, likely through the upregulation of the Wnt/β-catenin signaling pathway. This study elucidates the health benefits of *P. scandens* and paederoside in the prevention and treatment of osteoporosis.

## 1 Introduction

Osteoporosis represents a major disruption of bone homeostasis, predominantly affecting the elderly population, particularly postmenopausal women. The substantial adverse effects associated with currently approved anti-osteoporosis pharmacotherapies have limited their long-term clinical use (Authors Anonymous, 2021). In contrast, natural products, including tra-ditional Chinese medicines (TCMs), have been employed for millennia and have demon-strated anti-osteoporotic properties with minimal toxicity (Zhang et al., 2021). Elucidating the mechanisms of action and the material foundations of these bioactive TCMs is essential for the iden-tification of novel therapeutic targets and offers new perspectives for the development of anti-osteoporosis agents derived from natural sources.

*Paederia scandens* (Lour.) Merr. is a widely recognized edible wild botanical drug predominantly found in southern China and various Southeast Asian countries (Wang et al., 2014). This plant is rich in iridoids, flavonoids, feruloylmonotropeins, and volatile oils, which have been reported to possess anti-inflammatory, antioxidant, antirheumatic, analgesic, and hepatoprotective properties (Tang et al., 2022; Trung et al., 2023; Wu et al., 2019; Xiao et al., 2018; Xu et al., 2023; Zhang et al., 2022). Traditionally, *P. scandens* has been utilized in folk medicine for the treatment of jaundice, dysentery, and rheumatic pain (Wang et al., 2014). It is also commonly used as a culinary botanical drug in soups, with purported health benefits for bone development and general wellbeing (Panmei et al., 2019), thereby suggesting its potential utility in the prevention or treatment of osteoporosis and bone fractures. However, no indexed publications have reported on the anti-osteoporotic effects of *P. scandens* or its major metabolite paederoside. To the best of our knowledge, this is the first study to systematically investigate and demonstrate these effects, highlighting the novelty of our work.

The Wnt/β-catenin signaling pathway plays a crucial role in osteogenesis. Wnt pro-teins, functioning as extracellular ligands, bind to their respective receptors, leading to the inhibition of GSK-3β. This inhibition promotes the accumulation of β-catenin in the cytoplasm and its subsequent translocation to the nucleus, where it regulates the tran-scription of genes involved in cell proliferation and osteogenic differentiation (Gao et al., 2023; Zhang et al., 2023). Furthermore, activation of the Wnt/β-catenin pathway indirectly inhibits osteoclast dif-ferentiation and bone resorption by enhancing the secretion of osteoprotegerin (OPG), a decoy receptor that binds RANKL, a key factor in osteoclastogenesis (Gao et al., 2023; Zhang et al., 2023). Thus, the Wnt/β-catenin pathway, together with the OPG/RANKL axis, serves as a central regulator of bone homeostasis, wherein its activation enhances bone mass and strength, while its inhibition leads to bone loss and increased fragility (Gao et al., 2023). Despite the pharmacological potential of *P. scandens*, the effects of this botanical drug or its active metabolites on osteogenesis via modulation of the Wnt/β-catenin pathway remain largely unexplored.

The present study aims to investigate the anti-osteoporotic potential of *P. scandens* using an ovariectomized (OVX) rat model of osteoporosis. The active metabolites of *P. scandens* were predicted through network pharmacology approaches and qualitatively identified using HPLC-Q-TOF-MS. The anti-osteoporosis efficacy was evaluated both *in vivo* using the OVX rat model and *in vitro* in a co-culture system of osteoblasts and os-teoclasts. The phenotypes associated with osteogenesis and osteoclastogenesis were characterized through quantitative PCR, Western blotting, and immunohistochemical analyses. Furthermore, transcriptomic profiling via RNA sequencing was performed to elucidate the underlying mechanisms. The objective of this study is to clarify the phar-macological properties and material basis of *P. scandens* in the context of osteoporosis, thereby contributing to a deeper understanding of its therapeutic potential and facilitat-ing the identification of novel candidate metabolites for osteoporosis management.

## 2 Materials and methods

### 2.1 Materials

Paederoside (purity ≥99%, determined by HPLC) was acquired from Chengdu PufeiDe Biotech Co., Ltd. (Chengdu, China, JOT-11652). Specimens of *P. scandens* were obtained from Beijing Tong Ren Tang Co., Ltd. (Beijing, China), and a voucher specimen (desig-nated as No. 20211008) has been deposited in the botanical drugarium of the Dalian Municipal Central Hospital. Estradiol was procured from Shanghai Maclin Biological Reagent Co., Ltd. (Shanghai, China, E808987).

### 2.2 Preparation of *P. scandens* extract

*P. scandens* underwent two consecutive extractions using 95% ethanol, each lasting 2 h. The resulting solution was concentrated through evaporation, yielding a dry ex-tract, which was used in subsequent animal experiments. The content of paederoside in the crude botanical drug was quantified at 5.8 mg/g using HPLC.

### 2.3 Establishment of an ovariectomized (OVX) rat model

Female Sprague-Dawley (SD) rats, aged 6 months, were procured from Harbin Medical University. The experimental protocol was approved by the Animal Care Committee of Harbin Medical University (Approval No. SCXK 2020-0001). Rats were housed under standard conditions with *ad libitum* access to tap water and a standard laboratory diet (Hfkbio, Beijing, China).

The OVX rat model was established based on a previously reported protocol (Guo et al., 2023). Briefly, rats were anesthetized via intraperitoneal injection of sodium pentobarbital (30 mg/kg), followed by the excision of ovarian tissues. In the sham group, a minimal amount of adipose tissue adjacent to the ovaries was removed. To mitigate the risk of infection, penicillin and erythromycin ointments were applied to the incision sites. After a 3-month recovery period to allow for the development of osteoporosis, sham or OVX rats were randomly assigned to different treatment groups, i.e., groups treated with *P. scandens* extracts or paederoside. For the study involving *P. scandens* extracts (PS), rats were divided into three groups (n = 6 per group): sham, OVX, and OVX + PS (500 mg/kg). In the study involving paederoside, rats were divided to six groups (n = 6 per group): sham, OVX, positive control (estradiol, 10 μg/kg), and paederoside treatment at low (5 mg/kg), medium (10 mg/kg), and high (20 mg/kg) doses. Both *P. scandens* extracts and paederoside were dissolved in 10% (w/v) hydroxypropyl-β-cyclodextrin and administered orally for 12 weeks, whereas estradiol was administered via subcutaneous injection. At the end of treatment, rats were euthanized, and uterine tissues and blood samples were collected. Body weight gain and relative uterine wet weight were calculated. Serum calcium and phosphorus levels were analyzed using commercial assay kits (Nanjing Jiancheng Bioengineering Institute, Nanjing, China). Additionally, femoral bones were harvested for micro-computed tomography (micro-CT) and subsequent histological analyses including HE, Masson’s trichrome, and immunohistochemical staining.

### 2.4 Network pharmacology analysis

Network pharmacology analysis was performed following a previously reported protocol (Su et al., 2023). Potential active metabolites of *P. scandens* were identified using the TCMSP database (https://www.tcmsp-e.com/#/home), with screening thresholds set at oral bioavailability (OB) ≥ 30% and drug-like properties (DL) ≥ 0.18. Their canonical SMILES sequences were retrieved from PubChem (http://pubchem.ncbi.nlm.nih.gov). Targets interacting with these active metabolites were predicted using SwissTargetPrediction (http://www.swisstargetprediction.ch/) with a confidence probability ≥0.1. Disease-associated genes related to osteoporosis were retrieved from GeneCards (https://www.genecards.org), OMIM (https://www.omim.org/search/advanced/geneMap), DisGeNET (https://www.disgenet.com), and TTD (https://db.idrblab.net/ttd/) databases. Protein-protein interaction (PPI) networks were constructed using STRING (https://string-db.org), and network visualization and further analyses were performed using Cytoscape 3.10.1.

### 2.5 LC-QTOF-MS assays

Absorbable metabolites of *P. scandens* in 6 rats plasma were qualitatively analyzed using an Agilent 1200 HPLC system, an AB 3200 LC-ESI-MS system, and an AB Sciex X500R LC-QTOF-MS system. Metabolites separation via HPLC was carried out using an Agela Innoval ODS-2 column (4.6 × 250 mm, 5 μm) at a flow rate of 0.80 mL/min. The mobile phase comprised 0.03% (v/v) trifluoroacetic acid (solvent A) and methanol (solvent B). Gradient elution was programmed as follows: 10% solvent B from 0 to 10 min, transitioning to 100% solvent B from 45 to 55 min, and returning to 10% solvent B from 60 to 70 min. Detection of eluents was conducted at a wavelength of 230 nm. The eluent at 12.5 min was analyzed using ESI-MS and QTOF-MS. In the ESI-MS analysis, data were collected in both negative and positive ion modes, with a mass-to-charge ratio (m/z) range set between 200 and 800. For the QTOF-MS analysis, full-scan mass spectrometric data (MS) and product ion data (MS/MS) were obtained in positive ion mode within the same m/z range. All acquired data were processed using SCIEX OS software (SCIEX, v3.4).

### 2.6 RNA-seq and bioinformatics analysis of transcriptomes

Bone marrow cells were collected from OVX and OVX + PS (20 mg/kg) groups. Total RNA was extracted and used to construct cDNA libraries, which were then sequenced using the Illumina NovaSeq 6,000 platform. Analysis of differentially expressed genes (DEGs) was conducted using the DESeq2 R package (v1.20.0). PPI analysis of DEGs was performed via the STRING database and visualized using Cytoscape (v3.8.2) based on known interactions of the selected reference species.

### 2.7 Cell cultures

MC3T3-E1 and RAW264.7 cells were cultured in α-MEM medium (Gibco, Carlsbad, CA, United States) supplemented with 10% (v/v) FBS, 100 U/mL penicillin and 100 μg/mL streptomycin at 37 °C in humidified incubator of 5% (v/v) CO_2_.

### 2.8 Establishment of an osteoblasts/osteoclasts co-culture system

A co-culture system was established using Transwell inserts (0.4 μm pore size; Corning, United States). MC3T3-E1 cells were seeded in the lower chambers and RAW264.7 in the upper inserts, each at 5 × 104 cells per well. Cells were cultured in osteogenic medium containing 4 mM β-glycerophosphate and 25 μg/mL ascorbic acid to induce osteoblastic differentiation. Paederoside (0, 2, 5, 10 μM) was added into the medium to evaluate its effect on osteoblasts and osteoclasts. After 14 days of initial treatment, ALP staining (Beyotime, Nanjing, China) and TRAP staining (FUJIFILM Wako Pure Chemical Corporation, Japan) were performed to assess the number of osteoblasts and osteoclasts. Mineralization was assessed on day 21 using 2% Alizarin Red S staining (Aladdin, Shanghai, China).

### 2.9 Protein extraction and western blotting analysis

Femur bone tissue was collected and lysed in lysis buffer containing protease inhibitors (1% cocktail and PMSF) on ice for 2 h. Lysates were centrifuged at 12,000 g for 10 min at 4 °C to collect the supernatants. Proteins were separated by 10% SDS–PAGE and transferred onto polyvinylidene fluoride (PVDF) membranes. After blocking with 5% skim milk for 1 h, membranes were incubated overnight at 4 °C with primary antibodies, followed by incubation with HRP-conjugated secondary antibody for 1 h. Primary antibodies against BMP2, Smad1, Wnt3a, β-catenin, phospho-β-catenin, and RANKL were obtained from Proteintech (Wuhan, China), and those for Rnux2, Osterix, GSK-3β and phospho-GSK3β were from Abclonal (Wuhan, China). Bands were visualized using the Tanon 5200 ECL detection system (Tanon, China) and quantified using the ImageJ software (v1.54, NIH, United States).

### 2.10 qPCR assays

Total RNA was extracted from femur bone tissue, and cDNA was synthesized for qPCR using SYBR Green I kits (Takara, Japan). Gene expression levels of osteogenesis-related markers (ALP, Col1a1, Bglap, Osterix, and Runx2), osteoclastogenesis-related markers (NFATc1, Acp5, Ctsk, Mmp9), and Wnt pathway-related genes (Wnt3a, OPG, and RANKL) were normalized to housekeeping gene Gapdh. The primers used for qPCR assays are listed in Supplementary Material.

### 2.11 Histological staining (HE and Masson’s trichrome)

Femoral specimens were fixed in 4% paraformaldehyde for 48 h, decalcified in 10% EDTA solution (pH 7.4) at 4 °C for 4 weeks with gentle agitation, and then embedded in paraffin. Serial sagittal sections (5 μm) were prepared using a microtome (Leica RM2235, Germany). For hematoxylin–eosin (HE) staining, sections were deparaffinized, rehydrated, and sequentially stained with hematoxylin (5 min) and eosin (2 min). For Masson’s trichrome staining, sections were processed according to the manufacturer’s instructions (Solarbio, Beijing, China), with collagen fibers stained blue and cytoplasm stained red. Stained slides were examined using a light microscope (Olympus BX53, Japan).

### 2.12 Immunohistochemistry (IHC)

Paraffin sections (5 μm) were deparaffinized and subjected to antigen retrieval in 10 mM citrate buffer (pH 6.0) at 95 °C for 15 min. Endogenous peroxidase activity was blocked with 3% hydrogen peroxide for 10 min, followed by blocking with 5% bovine serum albumin (BSA) for 30 min at room temperature. Sections were incubated overnight at 4 °C with primary antibodies against BMP2 (1:200, Proteintech, Wuhan, China) and RANKL (1:200, Abclonal, Wuhan, China). After washing, sections were incubated with HRP-conjugated secondary antibody (ZSGB-Bio, Beijing, China) for 1 h at room temperature. Immunoreactivity was visualized using 3,3′-diaminobenzidine (DAB) substrate, and nuclei were counterstained with hematoxylin. Negative controls were treated with isotype-matched IgG instead of primary antibody. Images were acquired using a digital microscope (Olympus BX53, Japan). Quantitative analysis was performed by calculating the percentage of positively stained area and the integrated optical density (IOD) using ImageJ.

### 2.13 Statistical analysis

Statistical analysis was performed by Student’s t-test or one-way ANOVA with GraphPad Prism 8.0 (GraphPad Software, CA, United States). Data are presented as means ± standard error of the mean (SEM). A p-value <0.05 was considered statistically significant.

## 3 Results

### 3.1 *P. scandens* extract protected against osteoporosis in OVX rats

**FIGURE 1:**
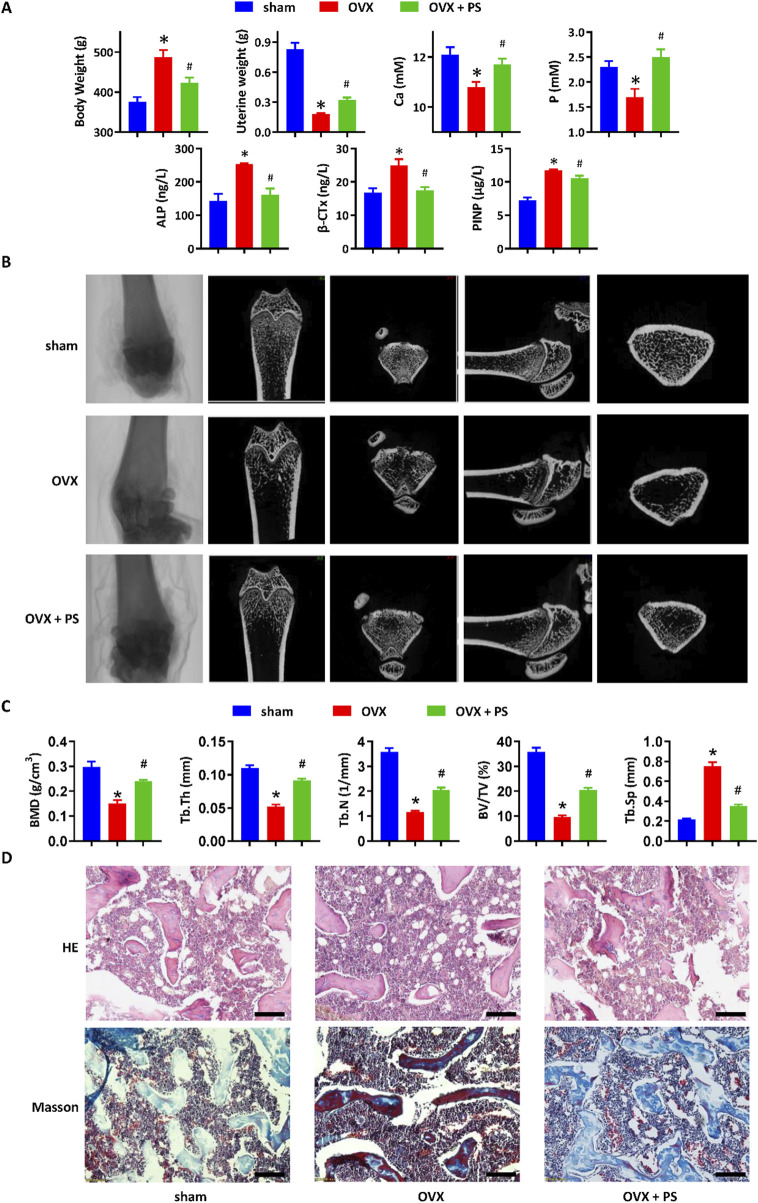
*P. scandens* protected against osteoporosis in OVX rats. **(A)** Effect of PS on the body weight gain, uterine wet weight, and serum levels of calcium, phosphorus, β-CTx, ALP, and P1NP in OVX rats treated with or without PS. **(B)** Representative micro-CT images of the femur bone in OVX rats treated with or without PS. **(C)** Bone microstructure parameters (BMD, Tb.Th, Tb.N, BV/TV, and Tb.Sp) of the femur bone according to micro-CT analysis. **(D)** HE and Masson staining of the femur bone in OVX rats treated with or without PS. Data are expressed as mean ± SD, n = 10. *, P < 0.05 vs. sham group. #, P < 0.05 vs. OVX group. PS, *P. scandens*. Scale bar = 50 μm.

The anti-osteoporotic efficacy of *P. scandens* was assessed using a rat model of osteoporosis induced by OVX. As shown in Figure 1A, 16 weeks post-OVX or sham surgery, OVX rats exhibited a notable increase in body weight, along with decreased uterine wet weight and reduced serum calcium and phosphorus levels. Treatment with *P. scandens* extract reversed body weight gain and restored uterine weight, while also normalizing serum calcium and phosphorus levels. Furthermore, *P. scandens* extract significantly reversed OVX-induced elevations in serum levels of osteoclast-related factor (β-CTx) and osteoblast-related factors (ALP and P1NP) (Figure 1A). Similarly, micro-CT analysis also revealed a significant loss of bone mass in the femur of OVX rats compared to rats in the sham group, which was markedly ameliorated by *P. scandens* extract treatment (Figure 1B). Quantitative analysis demonstrated significant reductions in bone mineral density (BMD), trabecular thickness (Tb.Th), trabecular number (Tb.N), and bone volume/total volume (BV/TV), along with an increase in trabecular separation (Tb.Sp) in OVX rats, confirming the occurrence of osteoporosis (Figure 1C). Moreover, histological examination revealed that femur bone from sham rats displayed a normal structure characterized by evenly distributed and densely arranged bone trabeculae, whereas OVX rats exhibited reduced trabecular density and increased adipose infiltration (Figure 1D). Treatment with *P. scandens* extract restored intramedullary trabecular bone structure to a nearly-normal reticular meshwork (Figure 1D) and enhanced bone mineralization in OVX rats (Figure 1D). Thus, *P. scandens* extract demonstrated significant protective effects against OVX-induced osteoporosis.

### 3.2 Paederoside identified as the primary plasma-absorbed metabolite after oral administration of *P. scandens*

**FIGURE 2:**
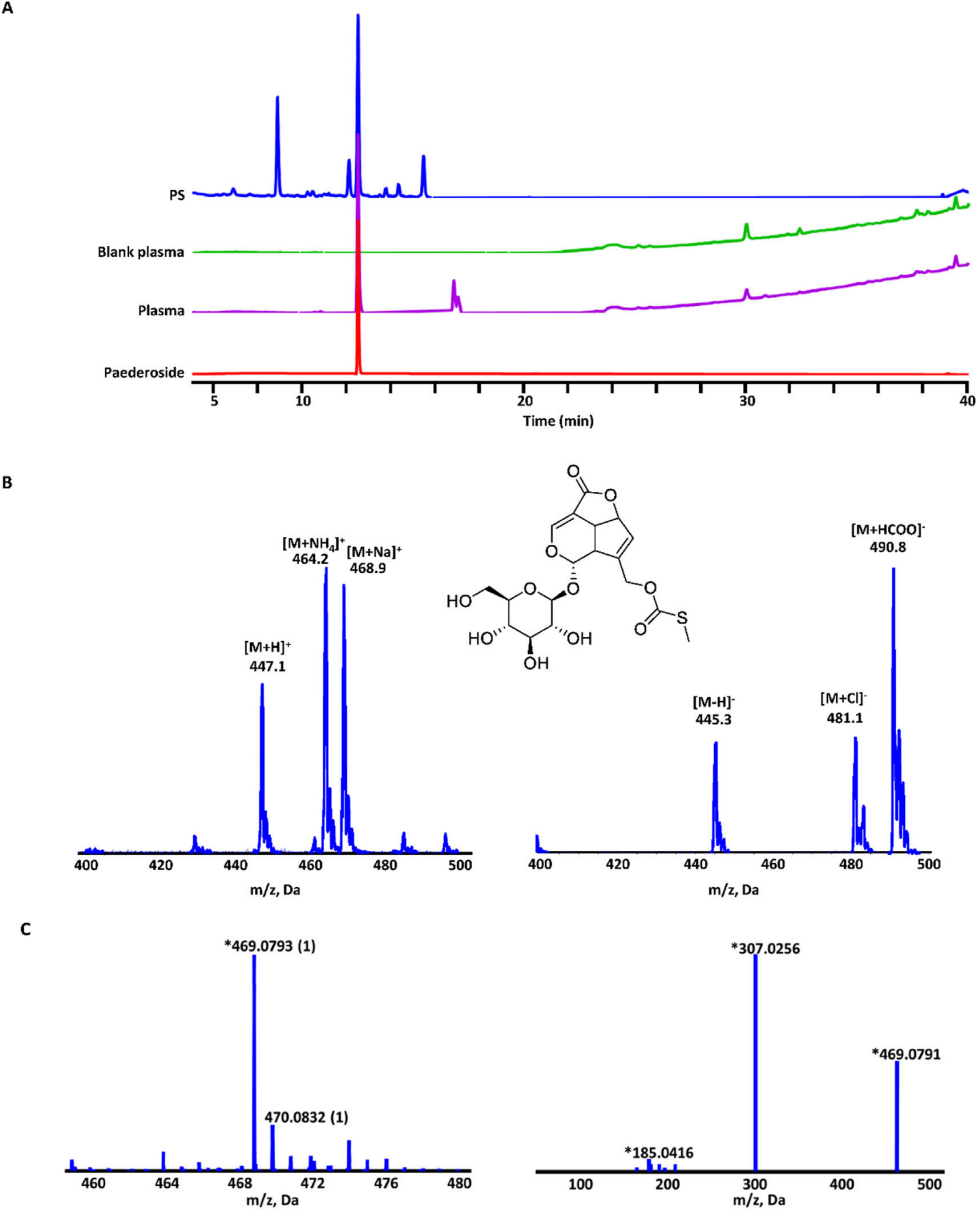
Paederoside was the main metabolite in rat plasma after oral administration of *P. scandens*. **(A)** Representative chromatograms of total extracts of PS, plasma samples from rats administered with or without PS, and Paederoside. **(B)** Q1 spectra of the main absorbable metabolite in rat plasma after oral administration of PS by LC-ESI-MS in positive (left) and negative (right) mode. **(C)** QTOF-MS (left) and QTOF-MS/MS (right) spectra of the main absorbable metabolite in rat plasma after oral administration of PS by QTOF-MS. PS, Paederia scandens.

To further elucidate the bioactive metabolites in the *P. scandens* extract, LC-QTOF-MS/MS was performed and 26 metabolites were identified, including iridoids, flavonoids, feruloylmonotropeins, and volatile oils (refer to Table 1). Subsequently, HPLC was performed using *P. scandens* extract, blank plasma, and plasma from rats administered the extract. The analysis revealed a distinct peak with a retention time of 12.48 min in rat plasma dosed with *P. scandens* extract, in comparison with the blank plasma (Figure 2A). This peak was further collected and analyzed using ESI-MS. In the Q1 spectra obtained from the ESI source, the primary absorbable metabolite produced precursor ions at m/z 447.1, 464.2, and 468.9 in positive ion mode, as well as m/z 445.3, 481.1, and 490.8 in negative ion mode (Figure 2B). QTOF-MS/MS was then performed with a standard paederoside sample used as the reference, the compound showed [M + Na]+ ion peaks at m/z 469.0789, and its MS/MS spectrum revealed characteristic fragment ions at m/z 307.0255 (Figure 2C). Based on the ion fragmentation pattern observed in MS2 and existing chemical references, the molecular formula was proposed as C18H22O11S, with a precision of 3.0 ppm. Furthermore, the retention time of the absorbable metabolite from *P. scandens* matched that of the standard reference, paederoside (Figure 2A). Consequently, paederoside was determined to be the principal metabolite absorbed into plasma following oral administration of *P. scandens* extract. Its role as an active metabolite was subsequently confirmed by functional studies.

**TABLE 1:** Identification of the metabolites in total extracts of *P. scandens* by LC-TOF-MS/MS.

No.	tR (min)	Formula	Molecula ion	Calculated mass (m/z)	Measured mass (m/z)	Error (ppm)	Identification
1	5.1	C15H11O5	M+	271.0601	271.0595	−2.2	Pelargonidin
2	5.1	C15H11ClO7	M+	303.0499	303.0497	−0.7	Delphinidin
3	6.0	C16H13O6+	M+	301.0707	301.0713	2.0	Peonidin
4	7.3	C18H24O12S	[M + Na]+	487.0881	487.0874	−1.4	Paederosidic acid
5	8.1	C19H26O12S	[M + Na]+	501.1037	501.1025	−2.4	Paederosidic acid methyl ester_qt
6	9.6	C18H24O12	[M + Na]+	455.116	455.1171	2.4	Asperulosidic acid
7	10.3	C16H22O11	[M + Na]+	413.1054	413.1042	−2.9	Scandoside
8	10.4	C17H24O10	[M + Na]+	411.1262	411.1267	1.2	Geniposide
9	10.5	C18H22O11S	[M + Na]+	469.0775	469.0766	−1.9	Paederoside
10	10.5	C18H22O11	[M + Na]+	437.1054	437.1068	3.2	Asperuloside
11	10.5	C16H20O10	[M + Na]+	395.0949	395.0940	−2.3	Deacetylasperuloside
12	10.5	C19H26O12S	[M + Na]+	501.1037	501.1022	−3.0	3,4-dihydro-3-methoxypaederoside
13	10.5	C19H26O12	[M + Na]+	469.1316	469.1319	0.6	Daphylloside
14	10.8	C28H32O14	[M + H]+	593.1865	593.1853	−2.0	Linarin
15	11.1	C21H20O12	[M + H]+	465.1028	465.1037	1.9	Quercetin 3-O-glucoside
16	11.2	C21H20O11	[M + H]+	449.1078	449.1092	3.1	Kaempferol 3-O-glucoside
17	11.8	C17H24O11	[M + Na]+	427.1211	427.1201	−2.3	6a-hydroxygeniposide
18	12.5	C26H30O14	[M + Na]+	589.1528	589.1546	3.1	10-O-E-feruloylmonotropein
19	12.7	C25H36O17S	[M + Na]+	663.1565	663.1580	2.3	6b-O-b-glucosylpaederosidic acid
20	13.6	C15H10O7	[M + H]+	303.0499	303.0503	1.3	Quercetin
21	14.5	C15H10O6	[M + H]+	287.0550	287.0550	0.0	Kaempferol
22	14.7	C15H10O4	[M + H]+	255.0652	255.0662	3.9	Diadzein
23	15.9	C16H12O5	[M + H]+	285.0757	285.0758	0.4	Acacetin
24	16.5	C36H46O19S	[M + Na]+	837.2246	837.2260	1.7	Saprosmoside E
25	16.7	C29H50O	[M-OH]+	397.3834	397.3843	2.3	Poriferast-5-en-3beta-ol
26	16.9	C29H50O	[M-OH]+	397.3834	397.3850	4.0	Beta-sitosterol

### 3.3 Paederoside promotes osteoblastogenesis and inhibits osteoclastogenesis *in vitro*

**FIGURE 3:**
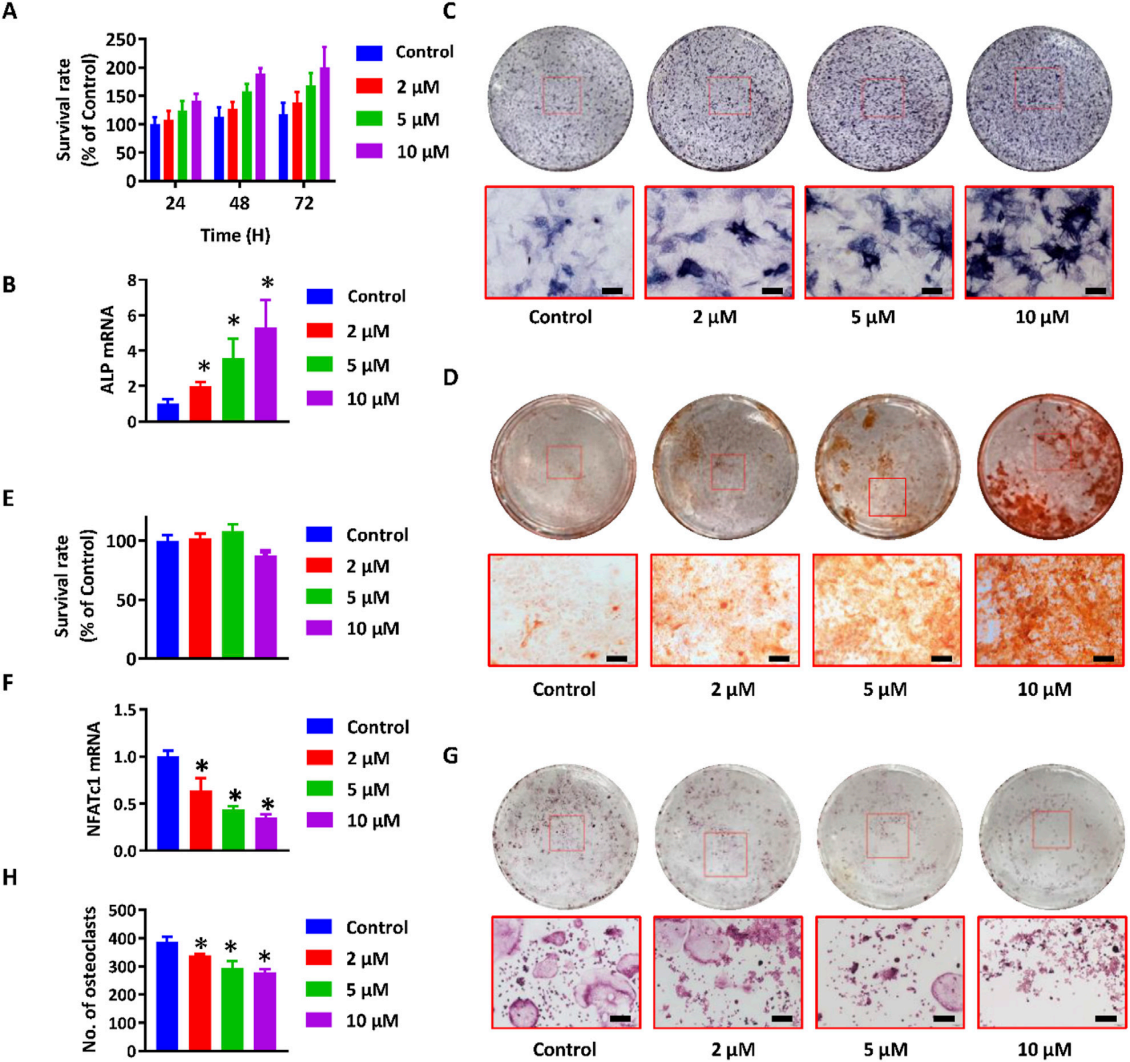
Paederoside promoted osteoblastogenesis and inhibited osteoclastogenesis in the osteoblasts and osteoclasts cells. **(A)** Proliferation of MC3T3-E1 cells after incubation of Paederoside (0–10 μM) for 24–72 h. **(B)** mRNA expression of ALP in MC3T3-E1 cells after incubation of Paederoside (0–10 μM) for 14 days. **(C)** ALP staining of MC3T3-E1 after incubation of Paederoside (0–10 μM) for 14 days (magnification, ×100). **(D)** Alizarin red staining of extracellular matrix mineralization of MC3T3-E1 cells after incubation of Paederoside (0–10 μM) for 21 days (magnification, ×100). **(E)** Proliferation of RAW264.7 cells after incubation of Paederoside (0–10 μM) in the osteoblasts and osteoclasts co-culture system. **(F,G)** TRAP staining and the number of TRAP (+) multinucleated osteoclasts generated from co-cultured RAW264.7 cells. **(H)** mRNA expression of NFATc1 in RAW264.7 cells after co-cultured with MC3T3-E1 cells in the presence of Paederoside (0–10 μM). Data was expressed as mean ± SD. *, P < 0.05 vs. the control group.

To validate the predictions from network pharmacology analysis and identify the major absorbable metabolites, an *in vitro* co-culture system comprising osteoblasts and osteoclasts was established utilizing MC3T3-E1 and RAW264.7 cells. This system has been frequently employed to concurrently assess the proliferation and mineralization of osteoblasts, as well as the differentiation of osteoclasts (Kim et al., 2021). Incubation with paederoside enhanced MC3T3-E1 cell proliferation (Figure 3A). Furthermore, the expression levels of ALP, the ratio of ALP-positive cells, and the area stained by Alizarin red significantly increased following Paederoside treatment (Figures 3B–D), indicating that paederoside facilitates both the proliferation and mineralization of osteoblasts. These findings indicate that paederoside promotes osteoblast differentiation and mineralization in MC3T3-E1 cells.

The ratio of osteoprotegerin (OPG) to receptor activator of RANKL is critical for maintaining bone homeostasis and plays a significant role in the osteoblastic regulation of osteoclastogenesis. Thus, differentiation of osteoclasts was evaluated using RAW264.7 cells co-cultured with MC3T3-E1 cells, both in the presence and absence of paederoside. CCK-8 assays demonstrated minimal cytotoxic effects on RAW264.7 cells (Figure 3E). In contrast, treatment with paederoside led to a concentration-dependent reduction in the number of TRAP positive multinucleated RAW264.7 cells (Figures 3F,G). Additionally, the mRNA expression of NFATc1, a key regulator of osteoclastogenesis, was also inhibited by paederoside treatment (Figure 3H). To further strengthen the reliability of these findings, we additionally confirmed the inhibitory effects of paederoside on osteoclast differentiation in primary bone marrow–derived osteoclasts, and the results (Supplementary Figure S1) were consistent with those obtained from the RAW264.7 cell model. Collectively, these findings indicate that paederoside promotes osteoblastogenesis while concurrently inhibiting osteoclastogenesis of osteoblasts and osteoclasts, respectively, within the co-culture system.

### 3.4 Paederoside alleviated osteoporosis in OVX rats

**FIGURE 4:**
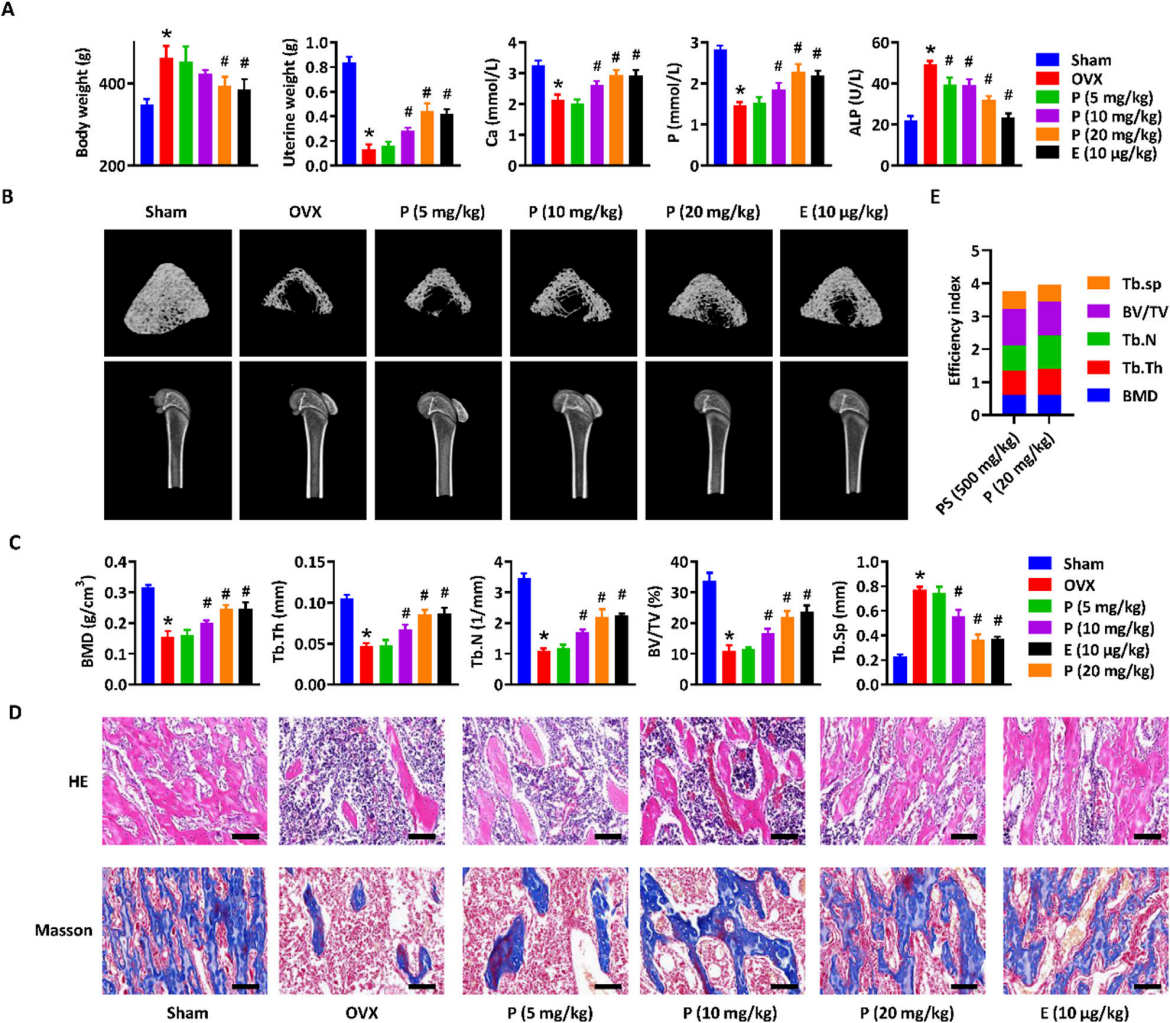
Paederoside alleviated osteoporosis in OVX rats. **(A)** Effect of Paederoside on the body weight gain, uterine wet weight, and serum levels of calcium, phosphorus, and ALP in OVX rats treated with or without Paederoside (5–20 mg/kg) or Estradiol (10 μg/kg). **(B)** Representative micro-CT images of the femur bone in OVX rats treated with or without Paederoside or Estradiol. **(C)** Bone density and biomechanical characteristics of the femur bone in OVX rats treated with or without Paederoside or Estradiol. **(D)** HE and Masson staining of the femur bone in OVX rats treated with or without Paederoside or Estradiol. **(E)** the efficiency index. It was calculated by summing up the fold changes of BMD, Tb.Th, Tb.N, BV/TV, and Tb. Sp after treatment of *P. scandens* (500 mg/kg) or Paederoside (20 mg/kg) in OVX rats. Data are expressed as mean ± SD, n = 10. *, P < 0.05 vs. sham group. #, P < 0.05 vs. OVX group. P, Paederoside; E, Estradiol. Scale bar = 50 μm.

To assess the anti-osteoporotic effects of paederoside, the osteoporosis phenotype was evaluated in OVX rats subjected to treatment with paederoside. Estradiol, a widely recognized pharmacological agent for the management of postmenopausal osteoporosis, served as a positive control in this study (Tella and Gallagher, 2014). Following a 12-week treatment regimen with paederoside at doses of 5, 10, and 20 mg/kg or estradiol at 10 μg/kg, body weight gain was accelerated, while uterine weight reduction was diminished in OVX rats compared to rats in the sham group (Figure 4A). Treatment with paederoside partially mitigated OVX-induced body weight gain and uterine weight loss. OVX rats also displayed significantly reduced calcium and phosphorus levels, alongside with increased serum ALP levels, indicative of an osteoporotic condition. These alterations in serum biomarkers were partially reversed following paederoside treatment (Figure 4A). Additionally, micro-CT analysis demonstrated substantial bone mass loss in the femur of OVX rats (Figure 4B), with significant declines in BMD, Tb.Th, Tb.N, BV/TV, Tb.Sp, confirming the onset of osteoporosis post-ovariectomy (Figure 4C). The bone mass loss was improved following paederoside treatment (Figures 4B,C). Furthermore, sham-operated rats exhibited evenly distributed and densely arranged trabecular bone in the femur (Figure 4D), whereas OVX rats displayed a reduction in trabecular bone and a low bone mass phenotype, which was restored to a dense trabecular network following paederoside treatment (Figure 4D). Paederoside also significantly inhibited OVX-induced bone resorption and collagen fiber loss, while promoting bone mineralization and formation in the femur (Figure 4D). Notably, estradiol treatment exerts similar effects comparable to those of paederoside regarding serum biochemical markers, as well as structural, morphological, and biomechanical properties of the femur. These results indicate that paederoside effectively alleviates osteoporosis in OVX rats, comparable to a well-known agent, estradiol. The therapeutic efficiency index was calculated by summing the fold changes in BMD, Tb.Th, Tb.N, BV/TV, and Tb. Sp following treatment with *P. scandens* (500 mg/kg) or paederoside (20 mg/kg) in OVX rats, yielding therapeutic efficacy indices of 3.76 and 3.96, respectively (Figure 4E). Considering its content (5.8 mg/g), paederoside was determined to contribute 15.3% to the therapeutic efficacy of *P. scandens*. These findings suggest that paederoside is a principal active metabolite responsible for the anti-osteoporotic activity of *P. scandens*. Additionally, HE staining of major organs (heart, liver, spleen, lung, and kidney) revealed no observable pathological alterations among treatment groups, indicating that paederoside did not induce systemic toxicity (Supplementary Figure S2).

### 3.5 Paederoside induced bone formation and inhibited osteoclast differentiation in OVX rats through modulating Wnt/β-catenin signaling pathway

**FIGURE 5:**
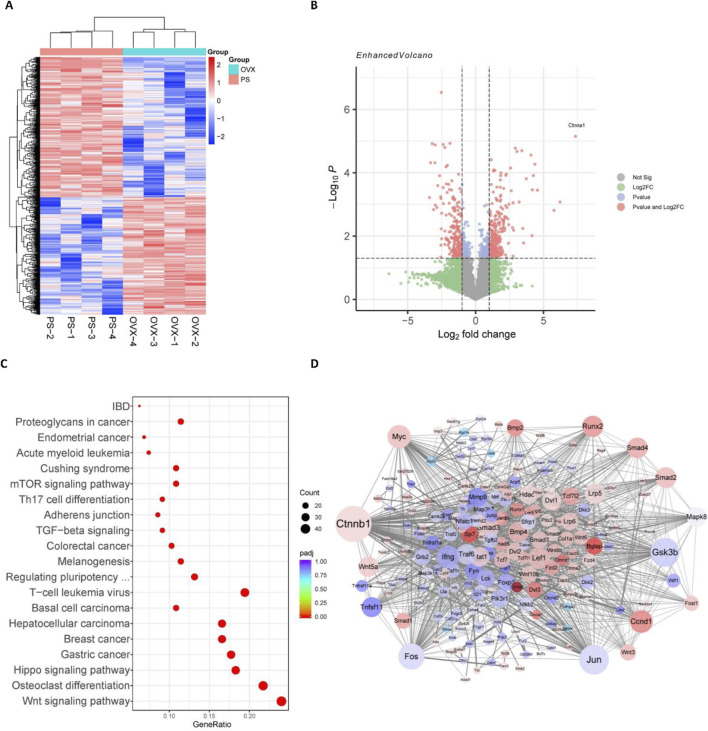
Wnt/β-catenin pathway was involved in the anti-osteoporosis of Paederoside by RNA-seq analysis. **(A)** Cluster analysis of differentially expressed genes (DEGs) after treatment of Paederoside. Colored bars indicate standardized log2 expression intensities. Red and blue represent upregulation and down-regulation, respectively. **(B)** Volcano plot of DEGs regulated by Paederoside treatment compared with OVX group. **(C)** Enrichment pathways according to the KEGG analysis. Dot colour represents padj value. Dot size represents the number of genes annotated to each KEGG term. **(D)** Protein-protein interactions (PPIs) network of DEGs regulated by Paederoside treatment.

In order to investigate the mechanism by which paederoside exerts its effects against osteoporosis, a transcriptomic analysis was conducted utilizing RNA sequencing (RNA-seq) on total RNA extracted from the bone marrow cells of OVX rats, either treated or not treated with paederoside at a dosage of 20 mg/kg. RNA-seq analysis revealed a significantly altered transcriptomic profile (Figure 5A), with 276 upregulated and 241 downregulated DEGs identified (|log2foldchange| > 1 and p-value <0.05) (Figure 5B). The top ten DEGs exhibiting the most significant changes included Tgfbr3, Ctnna1, Ifngr2, Lzts2, Rac1, Mmp9, Fosl2, Tcf7l2, Smad9, and Smad1. KEGG pathway analysis revealed significant alterations in pathways associated with the Wnt signaling pathway, osteoclast differentiation, and the Hippo signaling pathway (Figure 5C), all of which are known to play critical roles in the regulation of bone turnover. Furthermore, a PPI network was constructed to elucidate the interactions among the DEGs (Figure 5D). A total of 66 genes were identified as hub genes, each exhibiting a degree of connectivity greater than 20. Notably, Ctnnb1, Jun, Gsk3b, Fos, and Myc ranked highest, with degrees exceeding 50 (Figure 5D), underscoring their pivotal roles within the PPI network. Ctnnb1, which ranked the first and displayed the highest node degree of 95, could be a potential hub in mediating the effects of paederoside treatment. Ctnnb1 encodes the β-catenin protein, a key transcription factor within the Wnt/β-catenin signaling pathway. As a hub gene, Ctnnb1 demonstrated multiple interactions with other proteins, including Gsk3b, Myc, Runx2, and Smads, all of which are implicated in the Wnt/β-catenin pathway. These findings indicate that paederoside might confer protective effects against osteoporosis, potentially through the modulation of the Wnt signaling pathway.

**FIGURE 6:**
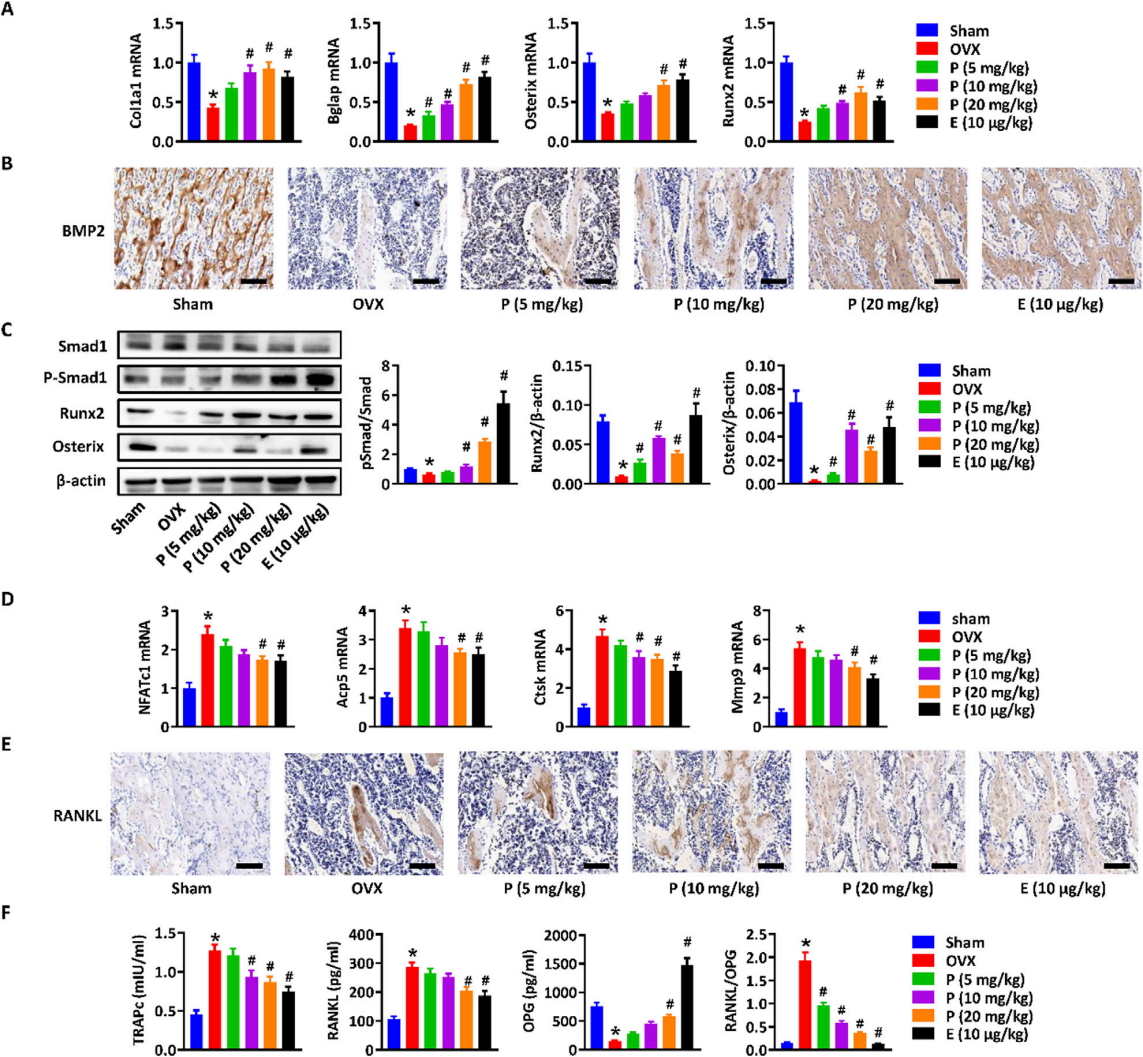
Wnt/β-catenin Paederoside activated osteoblastogenesis and inhibited osteoclastogenesis in OVX rats. **(A)** mRNA expression of osteogenesis-related markers (Col1a1, Bglap, Osterix, and Runx2) in the distal femurs of OVX rats treated with or without Paederoside (5–20 mg/kg) or Estradiol (10 μg/kg). **(B)** Immunohistochemical staining of BMP2 expression in the femur bone of OVX rats treated with or without Paederoside or Estradiol. **(C)** Protein expression and quantitative analysis of osteogenesis-related markers (Smad1, Runx2, Osterix) in the femur bone of OVX rats treated with or without Paederoside or Estradiol. **(D)** mRNA expression of osteoclastogenesis-related markers (NFATc1, Acp5, Ctsk, Mmp9) in the distal femurs of OVX rats treated with or without Paederoside. **(E)** Immunohistochemical staining of RANKL expression in the femur bone of OVX rats treated with or without Paederoside or Estradiol. **(F)** mRNA expression of TRAPc, RANKL, and OPG in the distal femurs of OVX rats treated with or without Paederoside. Data are expressed as mean ± SD, n = 10. *, P < 0.05 vs. sham group. #, P < 0.05 vs. OVX group. P, Paederoside; E, Estradiol. Scale bar = 50 μm.

To further elucidate the effects and mechanisms of paederoside on osteoporosis in OVX rats, we conducted a comprehensive assessment of bone formation and osteoclast differentiation mediated by the Wnt/β-catenin signaling pathway in OVX rats treated with or without paederoside using qRT-PCR. Initially, we observed that osteoblastic genes, such as Col1a1, Bglap, Osterix, and Runx2, were induced in a manner by paederoside when compared to the OVX control group (Figure 6A), similar to that of RNA-seq data. Given that Runx2 is a downstream target gene of β-catenin, these results further imply the involvement of the Wnt/β-catenin pathway in the action of paederoside. Additionally, BMP2, which interacts with the Wnt/β-catenin pathway through the SMAD protein family, was found to be downregulated in OVX rats relative to rats in the sham control. Following treatment with paederoside, the protein expression of BMP2 in the femoral bone was significantly increased (Figure 6B). Furthermore, paederoside treatment mitigated the OVX-induced suppression of Smad1 phosphorylation, a transcription factor downstream of the BMP2 signaling pathway. Correspondingly, the protein levels of Runx2 and Osterix, two critical transcription factors involved in osteoblast differentiation, were restored in the paederoside treatment group compared to the OVX control group (Figure 6C). Collectively, these findings indicate that paederoside promotes bone formation in OVX rats, potentially exerting its anti-osteoporotic effects through the Wnt and BMP2 signaling pathways.

Moreover, the expressions of osteoclastogenesis-related markers, including NFATc1, Acp5, Ctsk, and Mmp9, were elevated in bone marrow cells of OVX rats and could be restored by paederoside treatment (Figure 6D). RANKL, an essential cytokine for osteoclast formation, was significantly inhibited in the femur bone of OVX + PS group compared with OVX group (Figure 6D). The results suggested that paederoside might suppress differentiation of osteoclasts through the RANKL/RANK axis, further suggesting that paederoside exerts anti-osteoporosis effects possibly through induction of bone formation and inhibition of osteoclast differentiation in OVX rats involving the Wnt/β-catenin signaling pathway.

Immunohistochemical staining demonstrated that the elevated RANKL expression in the femoral bone of OVX rats was markedly suppressed by paederoside administration (Figure 6E). Furthermore, qRT-PCR analysis revealed that paederoside downregulated TRAPc and RANKL while upregulating OPG expression in the distal femurs of OVX rats (Figure 6F). These findings further confirm that paederoside inhibits osteoclast differentiation and activity, thereby reinforcing its dual role in promoting osteogenesis and suppressing osteoclastogenesis in OVX rats.

**FIGURE 7:**
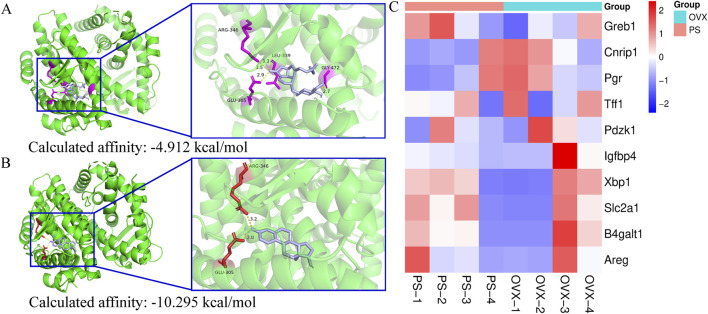
**(A)** molecular docking of estrogen receptor (3OLS) and paederoside by SwissDock; **(B)** molecular docking of estrogen receptor (3OLS) and Estradiol by SwissDock; **(C)** Transcriptional profiling of estrogen-responsive genes.

Molecular docking analysis revealed that paederoside could weakly bind to the ligand-binding domain of the estrogen receptor, exhibiting a relatively low binding affinity (−4.912 kcal/mol) (Figure 7A). The docking pose showed limited interactions, suggesting an unstable association with the receptor pocket. In contrast, estradiol readily occupied the same binding site, forming multiple hydrogen bonds with key residues such as ARG-346 and GLU-305, and displayed a markedly stronger binding affinity (−10.295 kcal/mol) (Figure 7B). These findings indicate that, unlike estradiol, paederoside does not exert its pharmacological effects through estrogen receptor binding, consistent with its non-estrogenic mode of action. Meanwhile, transcriptome assays indicated that estrogen-responsive genes, including GREB1, NRIP1, PGR, TFF1, PDZK1, IGFBP4, XBP1, SLC2A1, B4GALT1, and AREG, were not identified as differentially expressed genes (DEGs), further suggesting that paederoside may not act through estrogen-like mechanisms (Figure 7C).

### 3.6 Paederoside activates osteoblastogenesis and inhibits osteoclastogenesis via Wnt/β-catenin signaling pathways

**FIGURE 8:**
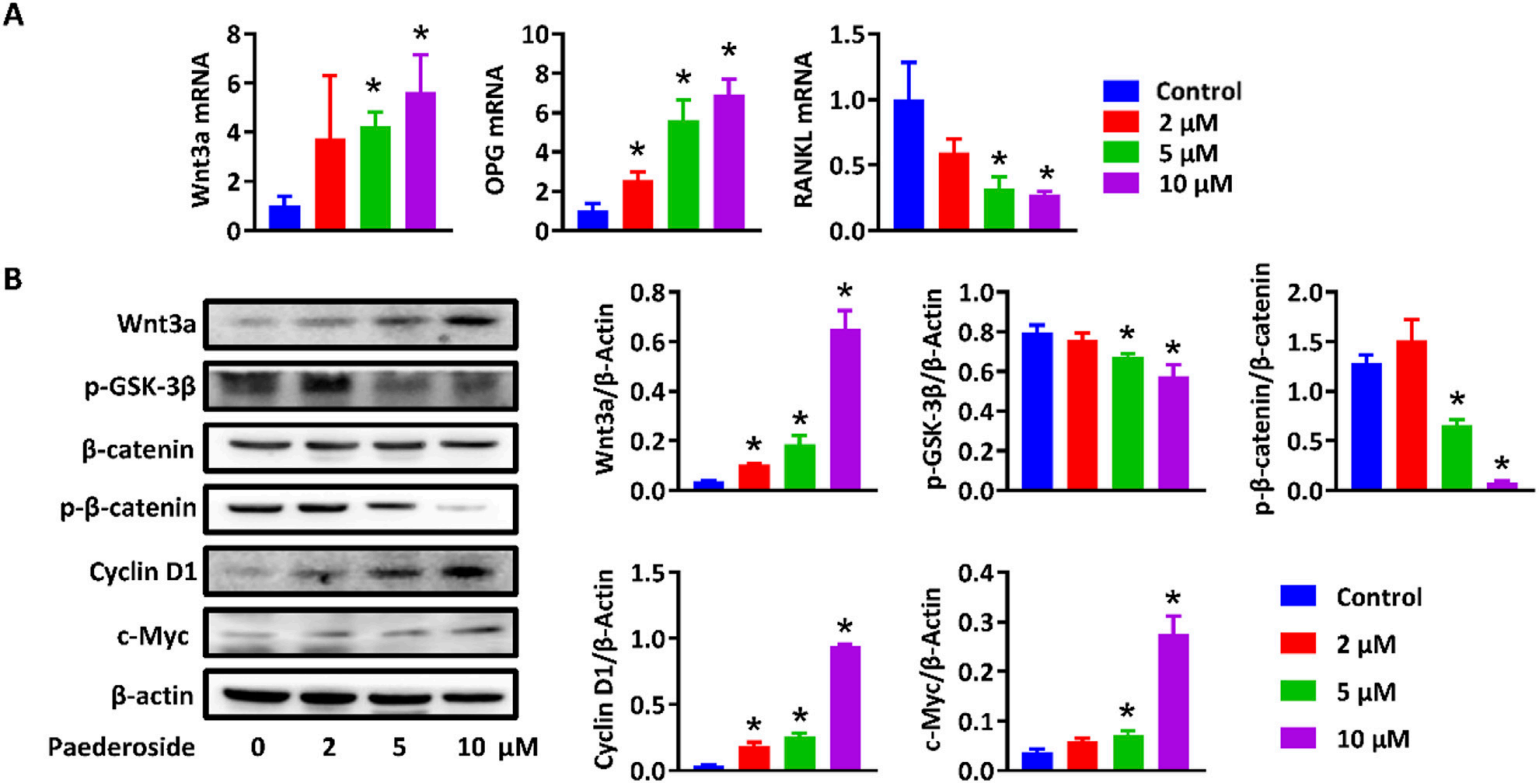
Paederoside promoted bone formation by regulating Wnt/β-catenin. **(A)** mRNA expression of Wnt3a, OPG, and RANKL in MC3T3-E1 cells after incubation of Paederoside (0–10 μM) for 14 days. **(B)** Protein expression and quantitative analysis of Wnt/β-catenin signaling in MC3T3-E1 cells after incubation of Paederoside (0–10 μM). Data was mean ± SD. *, P < 0.05 vs the control group.

Activation of the canonical Wnt/β-catenin signaling pathway in osteoblasts has been considered critical for osteogenesis and bone formation. To further verify the DEGs and activated Wnt/β-catenin signaling pathway, MC3T3-E1 and RAW264.7 cells were treated with or without paederoside. qRT-PCR showed that expression levels of Wnt3a and OPG in MC3T3-E1 cells were significantly upregulated after paederoside treatment (Figure 8A). Moreover, Western blotting revealed increased protein levels of Wnt3a and β-catenin, reduced phosphorylation of GSK-3β and β-catenin, and elevated Cyclin D1 and c-Myc expression in MC3T3-E1 cells (Figure 8B). These results further suggested that paederoside activate Wnt/β-catenin signaling pathways, which might be responsible for the induction of osteoblastogenesis in MC3T3-E1 cells.

## 4 Discussion

*P. scandens* is a widely recognized edible wild botanical drug known for its diverse pharmacological properties. In various regions of Asia, it is believed that cooking bone broth with *P. scandens* can enhance bone strength. However, the purported effects of *P. scandens* have not been sufficiently investigated. The present study aimed to assess the potential pharmacological effects and underlying material basis of *P. scandens* in combating osteoporosis, utilizing an OVX rat model and an *in vitro* co-culture system of osteoblasts and osteoclasts. The active metabolites responsible for the protective effects of *P. scandens* was identified via HPLC and the underlying mechanism were investigated through transcriptome analysis and subsequently validated *in vitro*. Our findings indicate that extracts of *P. scandens* confer protection against osteoporosis in OVX rats. Notably, paederoside, a principal iridoid glycoside found in *P. scandens*, was detected in rat plasma as a major absorbed metabolite, and its pharmacological relevance was demonstrated by significant anti-osteoporotic effects in OVX rats by promoting bone formation and inhibiting bone resorption. Transcriptomic analysis revealed substantial alterations in the Wnt/β-catenin signaling pathway following paederoside treatment in OVX rats. Furthermore, paederoside significantly stimulated the proliferation, differentiation, and mineralization of MC3T3-E1 cells, while concurrently reducing the ratio of TRAP-positive RAW264.7 cells and the expression of NFATc1 in the osteoblast-osteoclast co-culture system, through modulation of the Wnt/β-catenin and OPG/RANKL/RANK signaling pathways. These findings elucidate the health benefits associated with *P. scandens* and paederoside in the prevention and management of osteoporosis, indicating that paederoside holds promise as a potential candidate for the development of anti-osteoporosis pharmacotherapies.

### 4.1 *P. scandens* and its active metabolite Paederoside protected against osteoporosis in OVX rats

The urgent need for the development of novel therapeutic strategies for the management of osteoporosis in clinical settings has become increasingly apparent, despite significant progress in the formulation of new pharmacological agents for this condition. Natural products characterized by high efficacy and low toxicity present considerable advantages for long-term treatment and early prevention, particularly in light of the adverse reactions and toxicities associated with currently approved anti-osteoporosis medications (Lu et al., 2017; Martiniakova et al., 2020; Wang et al., 2023). In China, TCMs are extensively utilized for the treatment of osteoporosis and other orthopedic disorders, demonstrating established therapeutic efficacy. Various TCM formulations, individual botanical drugs, and active metabolites have been reported to possess significant anti-osteoporotic properties (Lei et al., 2021; Lin et al., 2017; Zhang et al., 2016). The present study evaluates the potential effects of *P. scandens* on osteoporosis in OVX rats. Our findings indicate that *P. scandens* provides a pronounced protective effect against OVX-induced osteoporosis in this rat model. In China and other Asian countries, *P. scandens* is traditionally employed as an edible wild botanical drug or medicinal plant for the treatment of bone fractures (Panmei et al., 2019). Additionally, it is commonly utilized for alleviating rheumatic pain (Wang et al., 2014; Xiao et al., 2018). To the best of our knowledge, this study represents the first evaluation and demonstration of the anti-osteoporotic effects of *P. scandens*. Given its long history of dietary and medicinal application, concerns regarding its safety are minimal. Therefore, *P. scandens* may serve as a therapeutic and preventive agent for individuals at high risk of developing osteoporosis.

The pronounced anti-osteoporotic effects of *P. scandens* prompted an investigation into its bioactive metabolites. Research has indicated that *P. scandens* possesses therapeutic potential for various conditions, including rheumatoid arthritis, gouty arthritis, hyperuricemia, acetaminophen-induced hepatic injury, and non-alcoholic fatty liver disease (Tang et al., 2022; Wu et al., 2019; Chen et al., 2022). Despite the diverse pathological mechanisms underlying these diseases, it is hypothesized that *P. scandens* exerts its effects primarily through the modulation of inflammatory responses and the reduction of oxidative stress (Wu et al., 2019; Xu et al., 2023). This suggests that similar active metabolites may be responsible for the multiple pharmacological activities attributed to *P. scandens*. Phytochemical analyses have identified iridoids, flavonoids, and volatile oils as the principal chemical metabolites of *P. scandens* (Wang et al., 2014). Previous studies have demonstrated that these metabolites exhibit anti-oxidative and anti-inflammatory properties (Wu et al., 2019; Xu et al., 2023), which may underpin the efficacy of *P. scandens* in combating osteoporosis. In the current study, we employed network pharmacology analysis to predict the potential active metabolites of *P. scandens* relevant to osteoporosis treatment, along with their associated targets. A variety of DEGs and corresponding pathways were identified, with particular attention given to CTNNB1 and osteoclast differentiation. To further elucidate the active metabolites, we utilized LC-TOF-MS/MS to identify the absorbed metabolites in the plasma of rats administered *P. scandens* extract orally. Our findings revealed that paederoside, an iridoid glycoside prevalent in *P. scandens*, was the primary metabolite detected in plasma, with a total iridoid content reaching 2% of the rats’ total body weight (Peng et al., 2015). This result aligns with previous studies reporting that iridoid glucosides, such as paederoside, paederosidic acid, methylpaederosidate, and Saprosmoside E (or Paederoside B) are the major absorbed metabolites detected in rat plasma after oral administration of *P. scandens* extracts. Our finding is also consistent with another study identifying paederoside, paederosidic acid, and paederosidic acid methyl ester as the predominant plasma metabolites, further confirming the reliability of our observations (Jiang et al., 2012; WANG et al., 2015). Iridoid glucosides derived from *P. scandens* have been shown to possess a range of pharmacological activities, including anti-inflammatory and immunomodulatory effects, analgesic properties, renoprotective effects against hyperuricemia-induced renal damage, hepatoprotective activity against CCl4-induced liver injury, and antiviral effects (Xu et al., 2023; Peng et al., 2015; Liu et al., 2012; Hou et al., 2014; Zhang et al., 2012). The significant presence of paederoside in plasma suggests its potential as the active metabolite responsible for the anti-osteoporotic effects of *P. scandens*. Indeed, our experiments demonstrated that paederoside mitigated osteoporosis in OVX rats, showing similar effects to those of estradiol, a clinically utilized estrogen medication for postmenopausal osteoporosis. Consequently, we conclude that paederoside is the principal active metabolite mediating the anti-osteoporotic activity of *P. scandens*.

### 4.2 Paederoside enhanced osteoblasts proliferation and inhibited osteoclasts differentiation through regulating Wnt/β-catenin and OPG/RANKL/RANK signaling

Osteoporosis is defined by a disruption in the equilibrium between bone resorption and bone formation. The pharmacological treatment of osteoporosis is designed to either diminish bone resorption or enhance bone formation. Consequently, established anti-osteoporosis medications are categorized into three groups: anti-resorptives, anabolic agents, and dual-action drugs (Reid and Billington, 2022). Recently, there has been significant interest in natural products derived from food and medicinal botanical drugs for their potential efficacy against osteoporosis, targeting various mechanisms. For instance, puerarin and glycyrrhizic acid have been shown to effectively inhibit osteoclastogenesis, osteoclast differentiation, and bone resorption (Xiao et al., 2020; Yin et al., 2019), whereas sanguinarine and ginsenoside Rg3 promote the proliferation and differentiation of osteoblasts, thereby stimulating bone growth (Zhang et al., 2018; Zhang et al., 2020). It is also commonly observed that some natural products, such as 7,8-dihydroxyflavone, quercitrin, and taxifolin, could simultaneously facilitate osteoblastic bone formation while inhibiting osteoclastic bone resorption (Satué et al., 2013; Xue et al., 2019). In this context, the pharmacological mechanisms underlying the protective effects of paederoside were investigated to provide valuable insights for the further development and application of *P. scandens* and paederoside as natural anti-osteoporosis agents. The present study demonstrates that paederoside not only promotes bone formation but also inhibits the differentiation of osteoclasts, indicating a dual action against osteoporosis.

**FIGURE 9:**
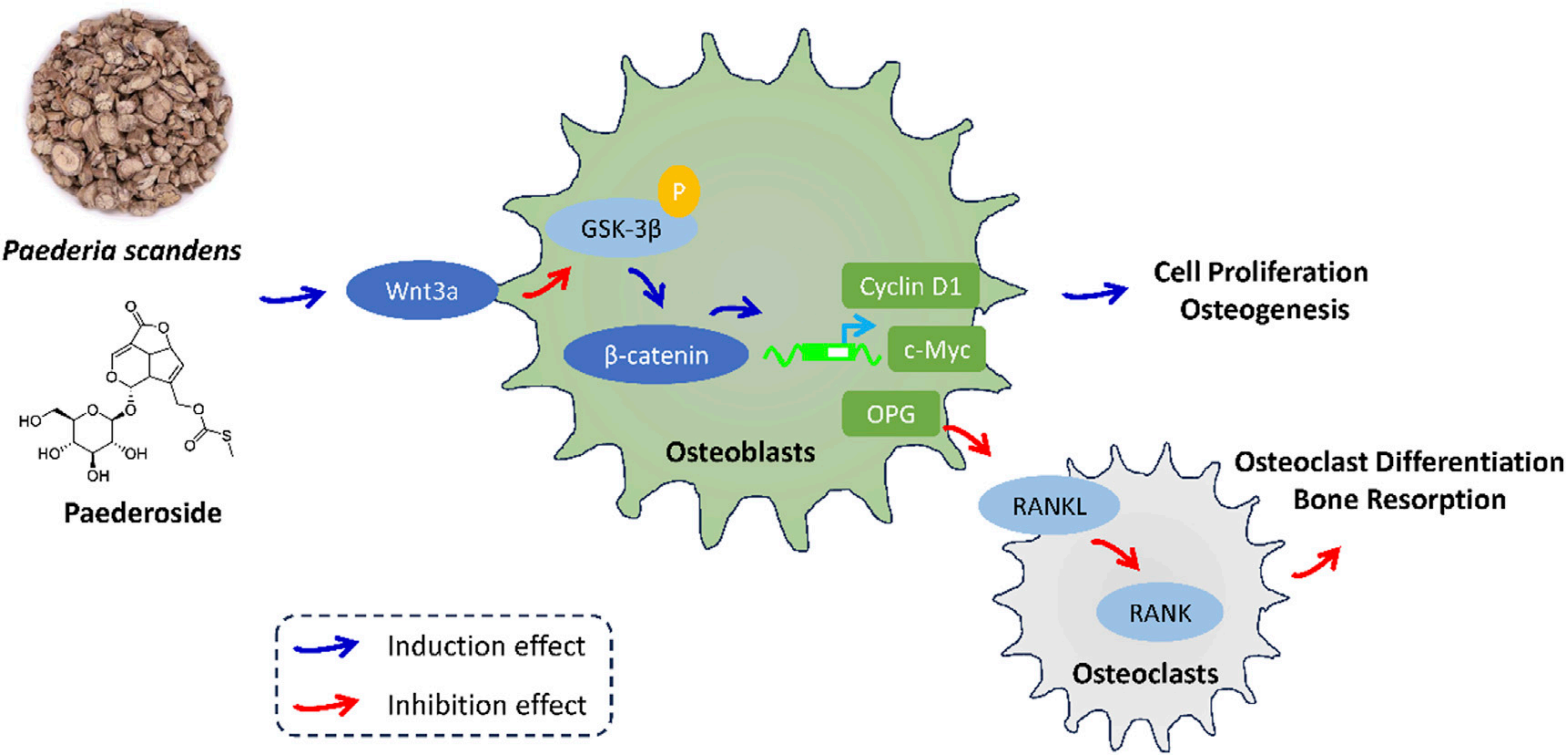
The effect and mechanism of *P. scandens* and Paederoside against osteoporosis.

The molecular mechanisms underlying bone formation and resorption have been the subject of extensive investigation. Significant signaling pathways that govern bone homeostasis have been identified and are being explored as potential pharmacological targets. In the current study, treatment with paederoside resulted in the upregulation of osteoblastic genes, including Col1a1, Bglap, Osterix, and Runx2, and downregulation of osteoclastogenesis-related genes, including NFATc1, Acp5, Ctsk, Mmp9, and RANKL, in OVX rats, suggesting activation of the Wnt signaling pathway and inhibition of the RANKL/RANK axis. Transcriptomic analyses indicated that DEGs associated with paederoside treatment were predominantly enriched in the Wnt signaling pathway and osteoclast differentiation, both of which have been deemed critical in maintaining bone homeostasis through the regulation of bone formation and resorption (Marini et al., 2023; Lu et al., 2023). The effects of paederoside on bone homeostasis were further corroborated in a co-culture system comprising pre-osteoblasts and pre-osteoclasts, which confirmed that paederoside promotes osteoblastogenesis while inhibiting osteoclastogenesis *in vitro*. Collectively, these findings provide compelling evidence that paederoside mitigates osteoporosis in OVX rats by modulating the Wnt/β-catenin and OPG/RANKL/RANK signaling pathways (Figure 9).

Currently, regulators of the Wnt/β-catenin signaling pathway, such as anabolic parathyroid hormone, teriparatide, and antibodies targeting the WNT antagonist sclerostin, have received approval for the treatment of osteoporosis, aimed at restoring bone strength in patients at risk of fractures (Baron and Kneissel, 2013; Gatti and Fassio, 2019). It is important to note that these therapeutic agents require daily subcutaneous injections, and in some instances, anabolic agents may simultaneously promote bone formation and the osteoblast-mediated secretion of RANKL, leading to bone resorption and potentially diminishing their beneficial effects on bone mass (Baron and Kneissel, 2013). In contrast, paederoside from *P. scandens*, presents minimal safety concerns and may enhance patient compliance. Additionally, treatment with paederoside over a 12-week period significantly improved bone mass, and no apparent adverse effects were observed during the study. Collectively, these findings suggest that paederoside is a promising candidate for the treatment of osteoporosis, characterized by high safety and efficacy.

The pivotal question that now confronts us is whether the promise observed in pre-clinical models can be successfully translated to human therapy. Looking ahead, the central challenge will be to leverage paederoside as a chemical starting point rather than as the final drug. Its current liabilities—poor oral bioavailability, rapid systemic clearance, and limited scalability—preclude direct clinical application. Instead, structure-guided medicinal-chemistry campaigns will be required to introduce strategic modifications that enhance pharmacokinetic properties (e.g., improved intestinal absorption, prolonged half-life), optimize physicochemical parameters (e.g., solubility, permeability), and ensure a favorable toxicological profile. Through iterative design-synthesize-test cycles, we anticipate the emergence of next-generation analogues that retain the core anti-osteoporotic activity of paederoside while overcoming its intrinsic limitations, ultimately delivering a clinically viable therapeutic for osteoporosis.

## 5 Conclusion

Paederoside, the principal active metabolite derived from *P. scandens*, exhibits protective effects against osteoporosis by simultaneously promoting osteogenesis while inhibiting osteoclastogenesis, primarily through modulation of the Wnt/β-catenin signaling pathway. These findings highlight the potential health benefits of *P. scandens*, with paederoside emerging as a promising natural metabolite candidate for the prevention and management of osteoporosis.

## Data Availability

The sequencing data generated in this study have been deposited in the NCBI Sequence Read Archive (SRA) under the accession number PRJNA1328381.
