# Supplementary figures and images for: Paederoside, an active metabolite of Paederia scandens, alleviates osteoporosis by modulating Wnt/β-catenin signaling
Source: Front Pharmacol. 2025 Sep 24;16:1670279. doi: 10.3389/fphar.2025.1670279 (PMC12504324; doi:10.3389/fphar.2025.1670279)

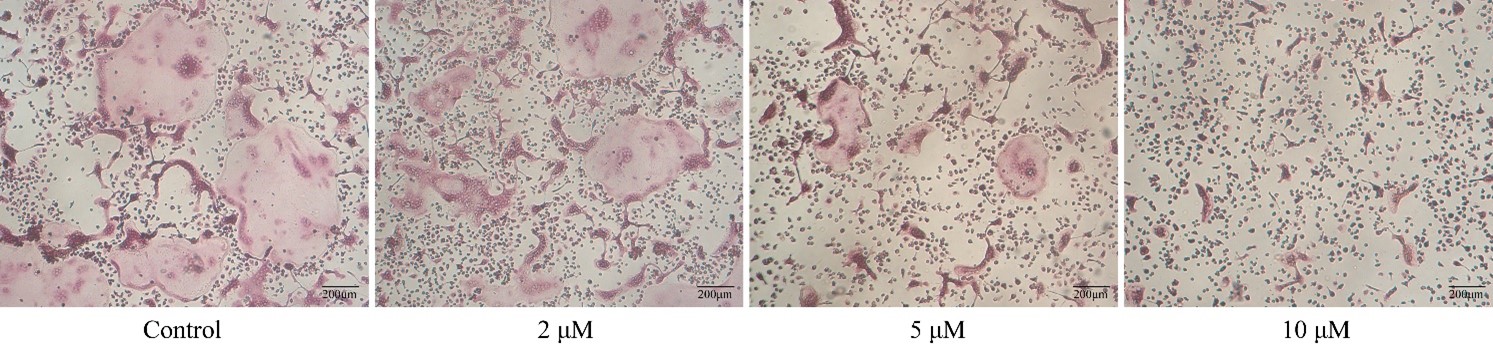

Supplement: Supplementary file 1 [file Image1.jpeg]

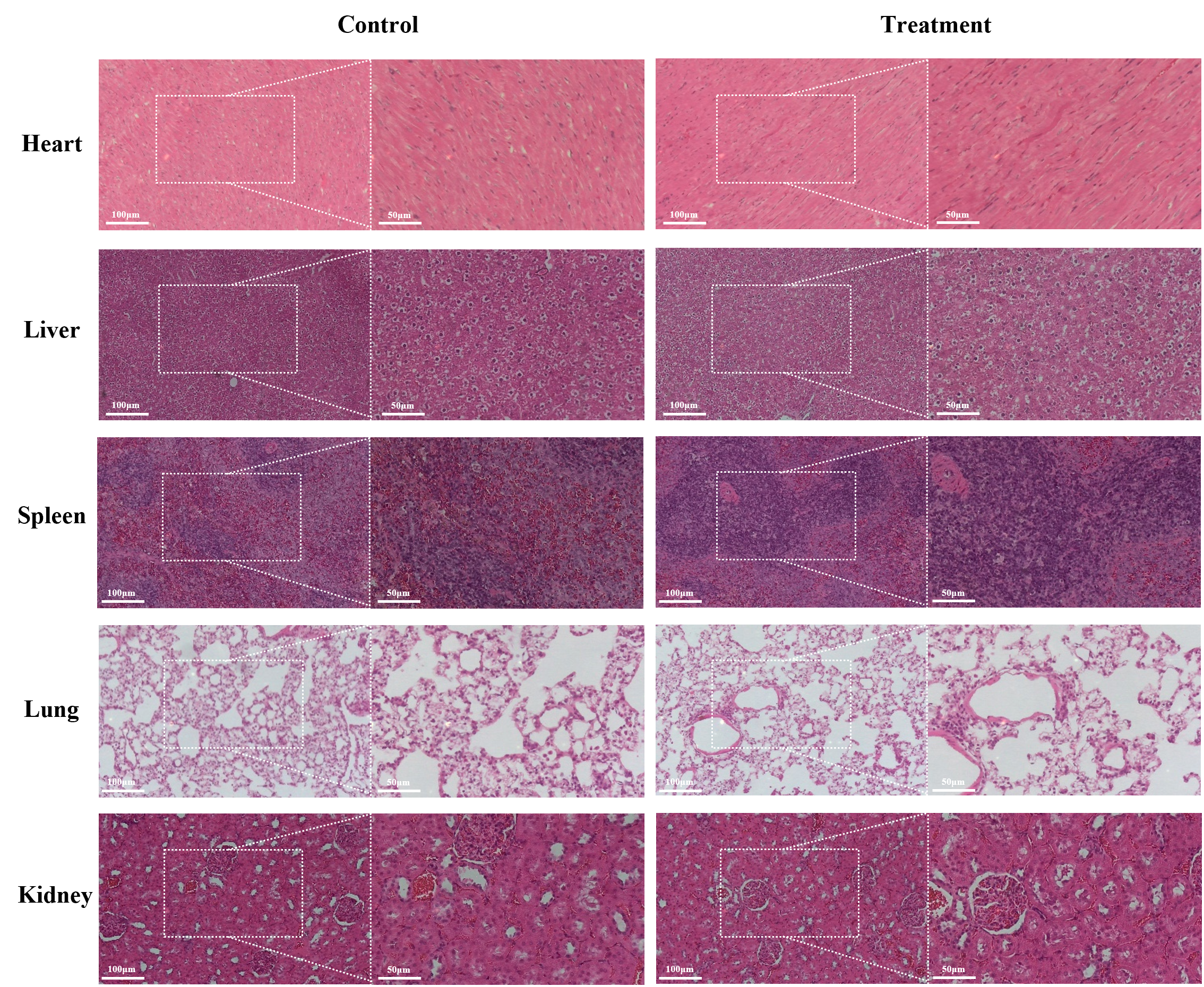

Supplement: Supplementary file 3 [file Image2.png]
