# Supplementary material for: Paederoside, an active metabolite of Paederia scandens, alleviates osteoporosis by modulating Wnt/β-catenin signaling
Source: Front Pharmacol. 2025 Sep 24;16:1670279. doi: 10.3389/fphar.2025.1670279 (PMC12504324; doi:10.3389/fphar.2025.1670279)
Supplement: Supplementary file 2 [file Table1.doc]

Table S1

| species | gene | forward: 5′-3′ | reverse: 5′-3′ |
| --- | --- | --- | --- |
| mice | Wnt3a | GATGGTGGTGGAGAAGCAC | GTGGGCACCTTGAAGTAGGT |
|  | OPG | TCCTGGCACCTACCTAAA | CACCTGAGAAGAACCCATC |
|  | RANKL | TGAAACTCACAGCCCTCT | TAAGCATCGGAATACCTC |
|  | NFATc1 | CAACGCCCTGACCACCGATAG | GGCTGCCTTCCGTCTCATAGT |
|  | ALP | CAGACCCTCCCCACGAGT | TGGATGTGACCTCATTGCCC |
|  | GAPDH | ACCACAGTCCATGCCATCAC | TCCACCACCCTGTTGCTGTA |
|  |  |  |  |
| rats | NFATc1 | GGCTACAGCCGCAGTAAATG | ACCAGATGTGGGTCCAGTTT |
|  | ALP | TGCAGGATCGGAACGTCAAT | GAGTTGGTAAGGCAGGGTCC |
|  | Runx2 | GCGTCCTATCAGTTCCCAAT | ATCAGCGTCAACACCATCAT |
|  | Bmp2 | CAGGTCTTTGCACCAAGATG | GCTGGACTTAAGACGCTTCC |
|  | COL1A1 | GGAGAGAGCATGACCGATGG | AAGTTCCGGTGTGACTCGTG |
|  | BGLAP | GCAGACCTAGCAGACACCAT | TTGGACATGAAGGCTTTGTCA |
|  | Osterix | GTCCTCTCTGCTTGAGGAAGAA | CTGTTGAGTCTCGCAGAGGG |
|  | Ctsk | GTATAACGCCACGGCAAAGG | TTCAGGGCTTTCTCGTTCCC |
|  | Runx2 | GCGGTGCAAACTTTCTCCAG | TGCAGCCTTAAATGACTCGG |
|  | Acp5 | TCTCTTCTACTGAGAGGTGCGA | AATCCCTGGTTCCGTGCTTT |
|  | GAPDH | TCAAGAAGGTGGTGAAGCAG | AGGTGGAAGAATGGGAGTTG |
